# Vision in two cyprinid fish: implications for collective behavior

**DOI:** 10.7717/peerj.1113

**Published:** 2015-08-04

**Authors:** Diana Pita, Bret A. Moore, Luke P. Tyrrell, Esteban Fernández-Juricic

**Affiliations:** Department of Biological Sciences, Purdue University, West Lafayette, IN, USA

**Keywords:** Collective behavior, Vision, Zebrafish, Golden shiner, Visual acuity

## Abstract

Many species of fish rely on their visual systems to interact with conspecifics and these interactions can lead to collective behavior. Individual-based models have been used to predict collective interactions; however, these models generally make simplistic assumptions about the sensory systems that are applied without proper empirical testing to different species. This could limit our ability to predict (and test empirically) collective behavior in species with very different sensory requirements. In this study, we characterized components of the visual system in two species of cyprinid fish known to engage in visually dependent collective interactions (zebrafish *Danio rerio* and golden shiner *Notemigonus crysoleucas*) and derived quantitative predictions about the positioning of individuals within schools. We found that both species had relatively narrow binocular and blind fields and wide visual coverage. However, golden shiners had more visual coverage in the vertical plane (binocular field extending behind the head) and higher visual acuity than zebrafish. The centers of acute vision (*areae*) of both species projected in the fronto-dorsal region of the visual field, but those of the zebrafish projected more dorsally than those of the golden shiner. Based on this visual sensory information, we predicted that: (a) predator detection time could be increased by >1,000% in zebrafish and >100% in golden shiners with an increase in nearest neighbor distance, (b) zebrafish schools would have a higher roughness value (surface area/volume ratio) than those of golden shiners, (c) and that nearest neighbor distance would vary from 8 to 20 cm to visually resolve conspecific striping patterns in both species. Overall, considering between-species differences in the sensory system of species exhibiting collective behavior could change the predictions about the positioning of individuals in the group as well as the shape of the school, which can have implications for group cohesion. We suggest that more effort should be invested in assessing the role of the sensory system in shaping local interactions driving collective behavior.

## Introduction

Animals in social groups often rely on the behavior of nearby conspecifics to obtain information about their environment (Dall et al., 2005). An individual’s ability to perceive and assess conspecific cues, such as the change in position and orientation of neighbors (Couzin et al., 2002; Couzin & Krause, 2003), is mediated by the sensory system (Dall et al., 2005) and may facilitate group cohesion. In some species, the use of both vision and the lateral line together allow an individual to assess important cues during schooling interactions. For example, in saithe (*Pollachus virens*) the lateral line is used to assess changes in the speed and direction of neighbors, while the visual system provides cues about neighbor position (Partridge & Pitcher, 1980). However, depending on the ecological context, some sensory dimensions may provide more reliable information about the surrounding environment. For example, social fish in visually obscure waters (e.g., fast-flowing, high sediment) place greater emphasis on the use of their chemosensory or mechanosensory (i.e., lateral line) systems to detect conspecifics (Pitcher & Parrish, 1993). However, in species inhabiting clear waters with low turbidity and readily available downwelling light, visual cues are spatially and temporally more reliable (Heuschele et al., 2009; Hogan & Laskowski, 2013).

Local interactions between group-mates drive the formation of large scale patterns of coordinated movement, referred to as collective behavior (Sumpter, 2010). In fish, a broad spectrum of collective behaviors has been characterized (Parrish, Viscido & Grünbaum, 2002). For example, fish that form groups to enhance foraging opportunities (i.e., shoals) are characterized by irregular neighbor separation and randomized individual alignment (i.e., low polarity) (Pitcher & Parrish, 1993). Whereas, in response to high predation risk, fish form more cohesive groups (i.e., schools), which tend to display fixed neighbor separation and ordered alignment (i.e., high polarity) (Pitcher & Parrish, 1993).

Although the behavioral mechanisms structuring these local interactions are still largely unknown (Lopez et al., 2012), individual-based models provide a useful way to predict collective interactions (Parrish, Viscido & Grünbaum, 2002; Sumpter, 2010). However, these models often make multiple sensory assumptions. For example, some models assume that individuals have a specific degree of visual coverage (Aoki, 1980), defined as the extent of the binocular plus the lateral visual fields, excluding the blind area at the rear of the head (Martin, 2007). In these models, visual coverage is generally assumed to be around 300°, limited by a 60° posterior blind area (Wood & Ackland, 2007). However, this estimate is based solely on a single species of whiting (Hall, Wardle & MacLennan, 1986) and may not accurately reflect other fish. Other models assume that individuals can perceive social cues up to a certain distance, which defines the individual’s social interaction range (Vicsek et al., 1995; Couzin et al., 2002). This interaction range is conventionally set to a radius of 0.5–2.0 body lengths (Barbaro et al., 2009; Katz et al., 2011) based upon empirical estimates solely from minnow schools (Partridge, 1980). Although these assumptions intend to consider some aspects of the sensory system, they do not necessarily reflect the configuration specific to the study species (Rountree & Sedberry, 2009; Romey & Vidal, 2013). This could be a major constraint in our ability to understand and model collective behavior, as recent evidence suggests that relaxing these sensory assumptions can influence model predictions (Lemasson, Anderson & Goodwin, 2009; Harpaz & Schneidman, 2014).

Our goal was to assess the visual system in two social species of cyprinids, the zebrafish (*Danio rerio*) and the golden shiner (*Notemigonus crysoleucas*), and discuss implications for their collective behavior. These species are excellent models because they both exhibit collective behavior and are thought to use vision as the primary modality during conspecific interactions (Miklosi & Andrew, 2006; Saverino & Gerlai, 2008; Polverino, Phamduy & Porfiri, 2013). We studied specific properties of the visual system that may be involved in the detection of conspecifics while animals are in schools: the visual field configuration (size of the binocular and lateral fields, and blind area), the density and distribution of retinal ganglion cells as well as eye size to estimate visual acuity, and the location of the center of acute vision.

The visual field configuration defines the visual coverage that an animal has around its head (Martin, 2007). Species with limited visual coverage (i.e., large blind areas) are expected to change their behavior (e.g., increase vigilance) to compensate for the lack of visual information in parts of their visual field (Guillemain, Martin & Fritz, 2002). Retinal ganglion cells relay information collected across the visual field to the visual centers of the brain (Pettigrew et al., 1988). Changes in the density of retinal ganglion cells are associated with changes in visual resolution across the retina (Collin, 1999). For instance, regions of the retina with a localized high density of retinal ganglion cells (i.e., center of acute vision) provide higher visual resolution. These centers of acute vision generally project into a specific region of the visual space, which may require an individual to move its body and modify its position within the group to detect changes in the behavior of group mates (Butler & Fernández-Juricic, 2014). In addition, eye size and the overall density of retinal ganglion cells can be used to estimate the upper limits of overall visual acuity (Land & Nilsson, 2012), which establishes the distances at which animals are expected to resolve conspecifics. For example, animals with higher visual acuity would detect changes in the behavior of conspecifics from farther distances (Fernández-Juricic & Kowalski, 2011), which may have consequences for the spacing of individuals within a group.

The foraging ecology of a species has been suggested as one of the main drivers in the evolution of visual systems in vertebrates (Collin, 1999; Martin, 2014). Both the zebrafish and golden shiner are social cyprinids found in well-lit freshwater environments (Sigler & Sigler, 1987; Spence et al., 2008). Both species, like many other cyprinids, are omnivorous visual feeders that forage on a variety of prey types located at the water surface (Sigler & Sigler, 1987; Spence et al., 2008). Based on the foraging strategies of the golden shiner and zebrafish, it is likely that both species possess visual configurations allowing them to efficiently detect and localize dorsally located, passive prey. We predicted that in an effort to reduce their vulnerability to predators, both species would have a reduced blind area, but intermediate binocular fields due to their omnivorous diets (Sigler & Sigler, 1987; Spence et al., 2008). In addition, these species are expected to have high acuity projecting to the surface of the water column to assist in the detection and capture of prey (Miyazaki, 2014). In fact, a previous study conducted on zebrafish characterized a ventro-temporal center of acute vision, which suggests adult zebrafish have high acuity in the region above the head (Mangrum, Dowling & Cohen, 2002), although this study did not estimate the specific projection into the visual field.

## Methods

We used 5 adult wildtype zebrafish (AB genetic strain) and 12 adult golden shiners from two commercial vendors. The golden shiners were purchased from a local bait fishery (Lafayette, Indiana, USA), while the zebrafish were obtained from The Zebrafish International Resource Center (ZIRC, Eugene, Oregon, USA). The zebrafish used for retina extractions also had their visual field measured prior to retina removal. For the golden shiners, we used different subjects for both the visual field measurements and retina extractions. Individuals were housed in separate aquaria under a 16-hour light: 8-hour dark cycle and supplied water via a flow-through filtration system. Fish were fed daily with a mixture of brine shrimp and commercially available dry foods (Tetramin^®^ Tropical Flakes). The Purdue Institutional Animal Care and Use Committee (1207000675) approved all experimental procedures.

### Visual field configuration

A variety of different approaches, both direct and indirect have been used to measure the visual field configuration of fish (Watanuki et al., 2000; Bianco, Kampff & Engert, 2011). However, direct estimates are often more precise, as they take into account the spatial extent of the retina and its projection into the visual space (Martin, 1984; Martin, 2007). For large species with large eyes that can tolerate supplemental ventilation and the placement of electrodes into the eye, the visual field can be measured with an electroretinogram on live animals (McComb & Kajiura, 2008; McComb, Tricas & Kajiura, 2009; Kajiura, 2010). However, for smaller species with small eyes, like the ones used in this study, ophthalmoscopic measurements are more appropriate (Collin & Shand, 2003). We measured the visual field configuration of 5 adult zebrafish and 9 adult golden shiners using an ophthalmoscopic reflex technique (Martin, 1984) and modified visual field apparatus developed for fish (Appendix S1). This technique requires the direct observation of the eye with an ophthalmoscope (Keeler Professional; Keeler Ophtalmic Instruments, Broomall, Pennsylvania, USA) to detect the light reflected from the retina, so observations were not made with the animal in water (Appendix S1). Individuals were measured immediately following euthanasia via rapid cooling through submersion in a 5 parts ice/1 part water mixture (Wilson, Bunte & Carty, 2009). The apparatus was configured such that 90° and 270° were directly in front and behind of the animal, respectively, along the horizontal plane, while 0° and 180° represented the vertical plane above and below the animal (Appendix S1). We recorded the visual field configuration across all angles around the head in 10° elevation increments. Our estimate of the visual field did not take into consideration variations due to eye movement, but considered the resting eye position, as previous studies suggest that the eyes assume a resting position postmortem (Martin, 1986). Additionally, the resting eye position is maintained in between convergent and divergent eye movements, and is thought to remain stabilized by the vestibulo-ocular reflex as the animal swims (Easter & Johns, 1974; Schairer & Bennett, 1986).

Based on the combined measurements of both the left and right eye at each elevation, we estimated the species’ binocular, lateral and blind area sectors (Martin & Katzir, 1999). The binocular field was determined separately at each elevation as the overlap in vision between both eyes while the blind area represented regions devoid of vision. For the lateral area, we applied the following equation: (360—(mean blind area + mean binocular field)/2) (Fernández-Juricic et al., 2008). Averages for each species were determined at each elevation with accuracy of ±0.5°.

### Retinal wholemounting

We extracted and wholemounted the retinas of 3 adult zebrafish and 3 adult golden shiners. Following euthanasia via rapid cooling (Wilson, Bunte & Carty, 2009), we submerged the head of the animal in Davidson’s Fixative for 3–5 h. While the eye remained attached to head by the optic nerve, we used spring scissors to remove the cornea, lens and vitreous humor. A dorsal cut was made to the retina before the eyecup was detached to ensure the correct orientation in later steps. We separated the retina from the eyecup by peeling away the sclera with forceps. The retina was then subjected to a (3%) bleaching solution until transparent to allow for the visualization of the ganglion cells. Following bleaching, 3–4 peripheral cuts were made to allow the retina to lie flat on a gelatinized glass slide. We followed the procedures described in Ullmann et al. (2012) to fix and stain the retina using a series of dehydrating and rehydrating steps in combination with 0.1% cresyl violet. In addition, we photographed images of the wholemounted retina and recorded area and perimeter measurements to account for retina shrinkage before and after staining using ImageJ (http://imagej.nih.gov/ij/). Average retina shrinkage values for the zebrafish and golden shiner were 0.07 ± 0.05 and 0.13 ± 0.07 respectively (mean ± SE).

Following staining, the retinas were viewed under an Olympus BX51 microscope. We traced the perimeter of the retinal periphery and optic nerve head with Stereo Investigator v.10 (MBF Bioscience, Williston, Vermont, USA) and then applied a sampling grid with a counting frame of 50 µm^2^ × 50 µm^2^ for a total of approximately 200 total sampling sites per retina. We then counted the total number of observed retinal ganglion cells at each of the sampling sites using ImageJ (http://imagej.nih.gov/ij/). Ganglion cells were distinguished from amacrine and glial cells according to previously established morphological characteristics (Hughes, 1977; Ehrlich, 1981; Collin & Pettigrew, 1988). We omitted counting sites where the retina appeared damaged or the cell visibility was poor. For the zebrafish, we counted 150 ± 54.9 sites per retina; while for the golden shiner we counted 112 ± 15.7 sites (mean ± SE). The Schaeffer coefficient of error for the zebrafish and golden shiner was 0.006 ± 0.0015 and 0.003 ± 0.0002 respectively (Glaser & Wilson, 1998). Topographic maps were generating using the R program “one cell map V8 svg version.R” (Garza-Gisholt et al., 2014). This program constructs isodensity maps from regions of localized cell density within a defined retinal outline created with Adobe Illustrator.

### Spatial resolving power

Retinal ganglion cells act as a bottleneck filter, summating the information from the other retinal cell types (i.e., bipolar cells, amacrine cells, photoreceptors) into a signal that is sent to the brain (Pettigrew et al., 1988). Considering the integral role that the ganglion cells play in relying visual information to higher processing centers, the anatomical estimates of spatial resolving power based on ganglion cell densities and eye size have been used extensively in the literature (Hughes, 1977; Collin & Pettigrew, 1989; Collin, 1999; Pettigrew & Manger, 2008). In addition, this anatomically derived measure of spatial resolving power has been shown to closely resemble behavioral estimates of visual acuity in species of fish, mammals and birds (Hughes, 1975; Hughes, 1977; Pettigrew et al., 1988; Pettigrew & Manger, 2008; Temple, Manietta & Collin, 2013).

In fish, the lens provides the primary source of focusing power due to the large dissimilarity in the refractive index between the aquatic environment and the optical media (Bone & Moore, 2008). The lens diameter also provides an index of eye size and body length, as it has been shown to increase in diameter as the fish matures (Pankhurst, Pankhurst & Montgomery, 1993). Consequently, calculations of spatial resolving power in fish incorporate lens dimensions (Collin & Pettigrew, 1989) as opposed to similar estimates in other vertebrates where eye axial length is generally used (Hughes, 1977; Martin, 1986). We followed (Collin & Pettigrew, 1988; Collin & Pettigrew, 1989) in the approach to estimate spatial resolving power using retinal ganglion cell density. For teleosts, the posterior nodal distance (*PND*) is calculated by multiplying 2.55 by the radius of the lens to estimate the area that an object would take up on the retina. Using the calculated *PND*, we then followed the equation from Pettigrew et al. (1988) and Williams & Coletta (1987) to estimate the retinal magnification factor, $R M F=\frac{2 \pi \ast P N D}{360}$ and anatomically derived spatial resolving power (*SRP*), where *D* is the density of retinal ganglion cells (cells/mm^2^):

\begin{eqnarray*} S R P=\frac{R M F}{2}\sqrt{\frac{2 D}{\sqrt{3}}}. \end{eqnarray*}

Using the estimated spatial resolving power of these cyprinid species, we calculate the extent of their ability to resolve objects in the environment. We followed the equation from Tyrrell et al. (2013) to estimate the threshold distance (*d*) at which an object exhibiting maximum visual contrast can be resolved under optimal light conditions, where *r* is the radius of the object and *α* is the inverse of spatial resolving power:

\begin{eqnarray*} d=\frac{r}{\tan \frac{\alpha }{2}}. \end{eqnarray*}

With this equation, it is possible to estimate the threshold maximum distance that an animal can resolve an object across various regions of the visual field. However, these distances do not incorporate the sensitivity and distribution of the photoreceptors, which also are also responsible for contrast sensitivity under different light environments (Lythgoe & Partridge, 1989). For the purposes of this study, we used the peak spatial resolving power (i.e., area with maximum ganglion cell density) and the minimum spatial resolving power (i.e., area with minimum retinal ganglion cell density) to calculate the maximum distance at which the body of conspecific could be resolved for both species in different regions of the visual field. In addition, we also estimated the maximum resolvable distance for the species-specific conspicuous markings, as it has been suggested that social fish use characteristics of the conspecific phenotype, such as spots or stripes as reference marks to assess changes in neighbor movements when schooling (Guthrie & Muntz, 1993; Bone & Moore, 2008). Wildtype zebrafish possess alternating light and dark horizontal stripes along the sides of their bodies, which may be used as a cue to assess the separable distances of group mates when shoaling (Rosenthal & Ryan, 2005). Golden shiners contrastingly do not have conspicuous stripes like the zebrafish; however, they do possess defined cycloid scales, which have smooth edges that could allow animals to distinguish individual scales. Therefore, we calculated the maximum resolvable distance for the golden shiner scales, as in some species the reflective scales act as a visual cue during social interactions (Rowe & Denton, 1997). We measured photographs of the zebrafish wildtype striping pattern (four individuals) digitally using ImageJ (http://imagej.nih.gov/ij/) and estimated the length of one cycle to be the height of two alternating, horizontal stripes, one dark and one light, assuming that both stripes were equal in dimension. Using similar procedures, we measured the average visible width of the cycloid scales of four golden shiners.

### Position and projection of the center of acute vision into the environment

We followed the protocol of Moore et al. (2012) to estimate the location of the center of acute vision on the flattened retina using Cartesian coordinates: -X, Y (dorsal nasal), X, Y (dorsal temporal), -X, -Y (ventral nasal), and X, -Y (ventral temporal). Coordinates were averaged across 3 individuals per species to determine the location of the center of acute vision along the four retinal axes (nasal, temporal, ventral and dorsal).

Additionally, using the monocular visual field of each eye and the ganglion cell topographic maps, we estimated the angular projection of the center of acute vision into the environment. Firstly, this required restoring the retina to its original shape because in the process of wholemounting we made several cuts to flatten it. The peripheral cuts of the topographic map were stitched together digitally using the R package, “Retistruct” (version 0.5.7) (Sterratt et al., 2013). The program uses a mathematical algorithm to digitally fit an object onto a spherical globe. The user provides the object of interest (i.e., retinal topographic map) and specifies the orientation in addition to other defining features of the retina, such as the optic disc (i.e., optic nerve head). We specified in the program the orientation of the dorsal cut applied to the retina during extraction. Following the reconstruction, we determined the angular position of the projection of the center of acute vision into the environment by reflecting the polar coordinates generated by the program into corresponding locations of each species’ visual field. These coordinates utilize a colatitude and longitude system where 0° lies at the center. The nasal and dorsal poles are −90° and the temporal and ventral poles are +90°.

## Results

### Visual field configuration

**Figure 1:**
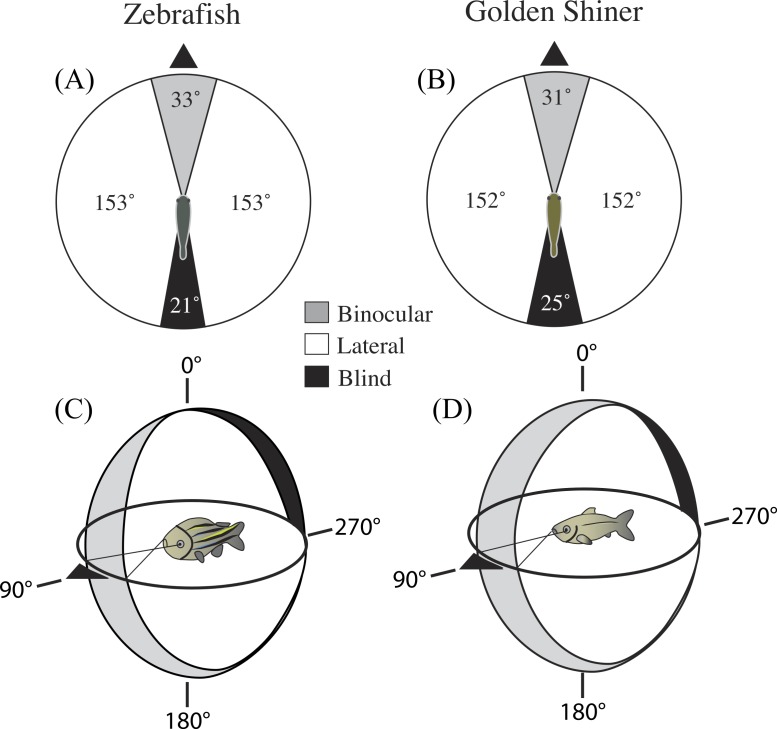
Visual field configuration of the (A) zebrafish and (B) golden shiner in the two-dimensional horizontal plane of the head (90°–270°). Three-dimensional depiction of the zebrafish (C) and golden shiner (D) visual field across all elevations measured about the head. The black triangle points anterior to the body of the fish in the horizontal plane. Both depictions represent the visual field while the eyes are at rest.

In the horizontal plane of the head (90°–270°), the binocular field width of the zebrafish and golden shiner were 33 ± 3.54° and 31 ± 1.67°, respectively (Figs. 1A and 1B). The height of the binocular field extended well above and below the horizontal plane of the head in both species (Figs. 1C and 1D). In the zebrafish, the upper portion of the binocular field projected to a point right above the head, 0° (Fig. 1C). However, in the golden shiner, the height of the binocular field extended an additional 40° behind the head (Fig. 1D), providing wider visual coverage in the vertical plane.

In the horizontal plane of the head, the zebrafish and golden shiner had a blind area of 21 ± 2.30° and 25 ± 1.20°, respectively. The size of the blind area limited the visual coverage (binocular plus lateral fields) in the horizontal plane to 339 ± 4.0° in the zebrafish and 335 ± 3.5° in the golden shiner. Considering all recorded elevations, the average visual coverage of these cyprinid species was: 336 ± 3.8° for the zebrafish, and 333 ± 3.5° for the golden shiner.

### Retinal ganglion cell density and centers of acute vision

**Figure 2:**
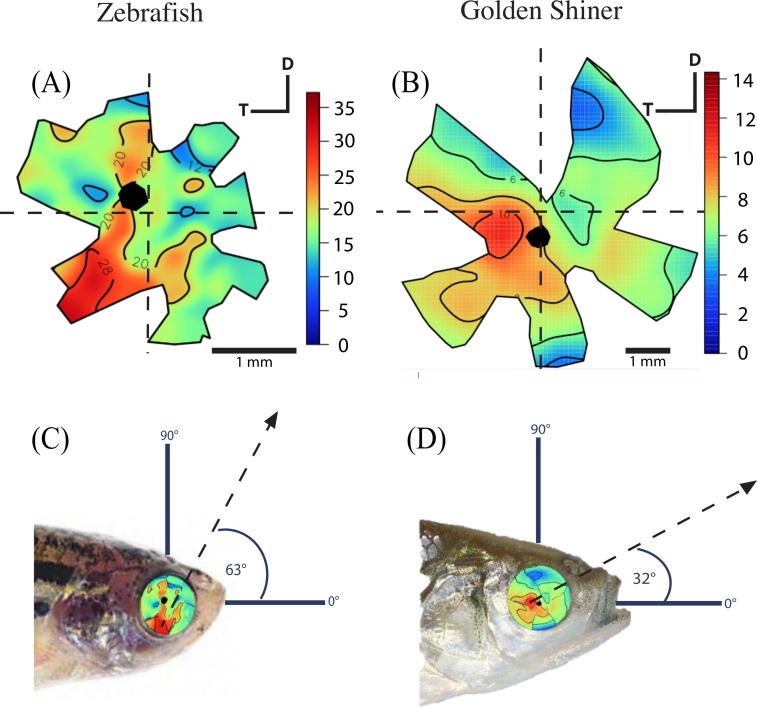
Retinal ganglion cell topography and projection of the center of acute vision. Ganglion cell topography across the retina of the (A) zebrafish and (B) golden shiner (cells/mm^2^ × 10^3^). Projection of the center of acute vision above the head of the (C) zebrafish and (D) golden shiner indicated with the dashed line and arrow. D, dorsal; T, temporal region.

**Figure 3:**
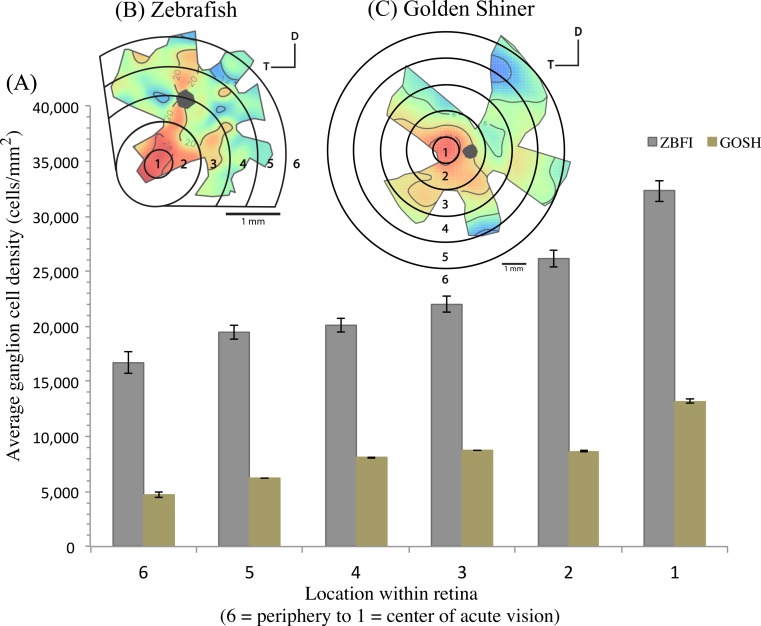
Variation in retinal ganglion cell density. (A) Graph depicting the variation in the retinal ganglion cell density across the retina within defined concentric regions from the periphery (6) to the center of acute vision (1). The bars represent the average retinal ganglion cell density ±SE in cells/mm^2^ for the (B) zebrafish (ZBFI) and (C) golden shiner (GOSH).

The density of retinal ganglion cells was not homogeneous across the retina (Fig. 2). Both species had a high density of retinal ganglion cells in the ventro-temporal region of their retinae and the pattern of cell density increase was concentric (Fig. 2). The characteristics of these areas of high retinal ganglion cell density (i.e., lack of morphological features associated with retinal invagination on the wholemounted retina, concentric increase in cell density) suggest that the centers of acute vision of both species are *areae* (i.e., enlargement of the retinal tissue due to the high density of photoreceptors and retinal ganglion cells; Walls, 1942). The zebrafish had a peak ganglion cell density of 36,224 ± 730 cells/mm^2^, while the golden shiner a peak density of 14,380 ± 1272 cells/mm^2^. In order to quantify the change in cell density across the retina, we utilized a concentric sampling approach to best reflect the characteristics of the *area* (Figs. 3B and 3C). The cell density difference between the center of acute vision (i.e., maximum density) and the retinal periphery (i.e., minimum density) was 18.29 ± 2.41% in the zebrafish and 17.55 ± 2.86% in the golden shiner on average (Fig. 3A).

Based on the ventro-temporal location of the centers of acute vision (Figs. 2A and 2B), we can assume that both species possess high acuity vision in the fronto-dorsal region of their visual field (Figs. 2C and 2D). For the zebrafish, the center of the *area* projected to −32 ± 15°, −64 ± 10° (nasal, dorsal) of the visual field while the center of the *area* projected to −20 ± 8°, −9 ± 4° (nasal, dorsal) of the visual field in the shiner. However, the location of centers of acute vision on the retina was slightly different in both species (Figs. 2C and 2D). For the zebrafish, the center of the *area* was more ventrally shifted and located −0.10 ± 0.06 along the nasal-temporal axis and −0.21 ± 0.11 along the dorso-ventral axis. In the golden shiner, the center of the *area* was more temporally shifted and located −0.01 ± 0.12 along the nasal-temporal axis and −0.17 ± 0.04 along the dorso-ventral axis. Consequently, the fronto-dorsal projection of the area varied between species.

### Spatial resolving power and maximum resolvable distance

In terms of eye measurements, we recorded the average lens diameter (mm) of both species as 0.83 ± 0.03 for the zebrafish and 2.00 ± 00 for the golden shiner; the eye transverse diameter was 1.91 ± 0.04 and 4.53 ± 0.20; and the eye axial length was 1.35 ± 0.10 and 3.20 ± 0.23, respectively. Additionally, the average total body length (cm) was 4.17 ± 0.07 for the zebrafish, and 7.4 ± 0.06 for the golden shiner.

According to the peak density of retinal ganglion cells and lens size, the visual acuity of the zebrafish and golden shiner were estimated to be 1.89 ± 0.02 and 2.86 ± 0.12 (cycles/degree), respectively. With their highest acuity, provided by the center of acute vision, zebrafish could resolve the body a conspecific up to 75.80 cm (18 body lengths) away and the striping pattern up to 19.49 cm (5 body lengths) away, while golden shiners could resolve a conspecific up to 2.92 m (39 body lengths) away and the cycloid scales of a conspecific up to 19.66 cm (3 body lengths) away. In the peripheral region of the retina with the lowest cell density, visual acuity was estimated as 0.08 ± 0.06 cycles/degree for the zebrafish, and 1.18 ± 0.15 cycles/degree for the golden shiner. Based on this retinal periphery estimates, a zebrafish could resolve a conspecific and the striping pattern up to 32.08 cm (8 body lengths) and 8.25 cm (2 body lengths), respectively; while the golden shiner could resolve a conspecific and the cycloid scales up to 1.20 m (16 body lengths) and 8.11 cm (1 body length), with the periphery of the retina.

## Discussion

Our results show that both cyprinid species share similar visual system properties: a wide degree of visual coverage and a ventro-temporally located center of acute vision projecting fronto-dorsally, which seems to be an *area*. However, there are some between species differences in the height of the binocular field, spatial resolving power, and the specific projection of the center of acute vision into the visual field. Additionally, our study is the first to estimate the spatial resolving power of adult zebrafish and golden shiners anatomically, using retinal ganglion cell densities. Although our estimate of the visual acuity in adult zebrafish (i.e., 1.89 cycles per degree) was higher compared to behaviorally measured estimates using the optokinetic test (i.e., 0.6 cycles per degree) (Tappeiner et al., 2012; Cameron et al., 2013) this dissimilarity may be related to the additional filtering of information that occurs in the brain (Hughes, 1977; Pettigrew & Manger, 2008), and the fact that the optokinetic response relies on a specific subset of ganglion cells, and may not accurately reflect the total cell population (Pettigrew et al., 1988).

There has been limited visual field data collected on fish. Past studies have focused on predatory species belonging to the Batoidea, Serranidae and Lepisosteidae Families (Collin & Northcutt, 1995; Collin & Shand, 2003; McComb & Kajiura, 2008). Our study is the first to characterize the visual fields of two members of the Cyprinidae, the largest Family of freshwater fish. In both cyprinid species, the binocular field widths are reduced in comparison to predatory species like the coral trout, painted comber, and blacktip grouper, which have binocular fields 36°, 40°, 54° wide, respectively (Collin & Shand, 2003). The reduced binocular field widths of the zebrafish and golden shiner are likely associated with their foraging behavior focused on catching sessile and slow moving prey. However, the lateral fields of both species are relatively wider than that of some members of the Lepisosteidae Family (137° in the horizontal plane; Collin & Northcutt, 1995), which are common predators of zebrafish and golden shiners (Schultz, 2004; Spence et al., 2008). Wide lateral fields in these two cyprinids may provide greater visual coverage to detect predators as well as conspecifics when schooling.

Variation in retinal topography has been associated with the foraging ecology and habitat structure (Collin & Pettigrew, 1988). Previous studies in other schooling cyprinid species, such as the sardine (*Sardinops melanostictus* and *Etrumeus sadina*) and anchovy (*Engraulis japonicus*), found a ventral-temporal *area* projecting upward into the frontal visual field (Tamura & Wisby, 1963; Pankhurst, 1989; Miyazaki, 2014). The overall location of the *areae* is similar to that of the zebrafish and golden shiner, likely due to their similar foraging ecology (i.e., dorsally located prey). However, we found between-species differences in the projection of the *areae*, which may be related to their relative position in the water column. Zebrafish inhabit shallower waters than golden shiners, which may increase their susceptibility to aerial predators (Keast & Fox, 1992; Spence et al., 2008; Luca & Gerlai, 2012). Therefore, zebrafish may align their center of acute vision with the area of the environment where attacks from aerial predators are more likely, due to its more dorsal projection. It is possible that the selection pressure relative to aerial predators is not as pronounced in golden shiners because they are deeper in the water column.

### Implications for collective behavior

Based on the characteristics of the visual field configuration and retinal topography reported in this study, we developed species-specific predictions relative to their collective behavior (i.e., positioning, orientation and spacing of individuals in a group) that can be tested empirically in the future.

**Figure 4:**
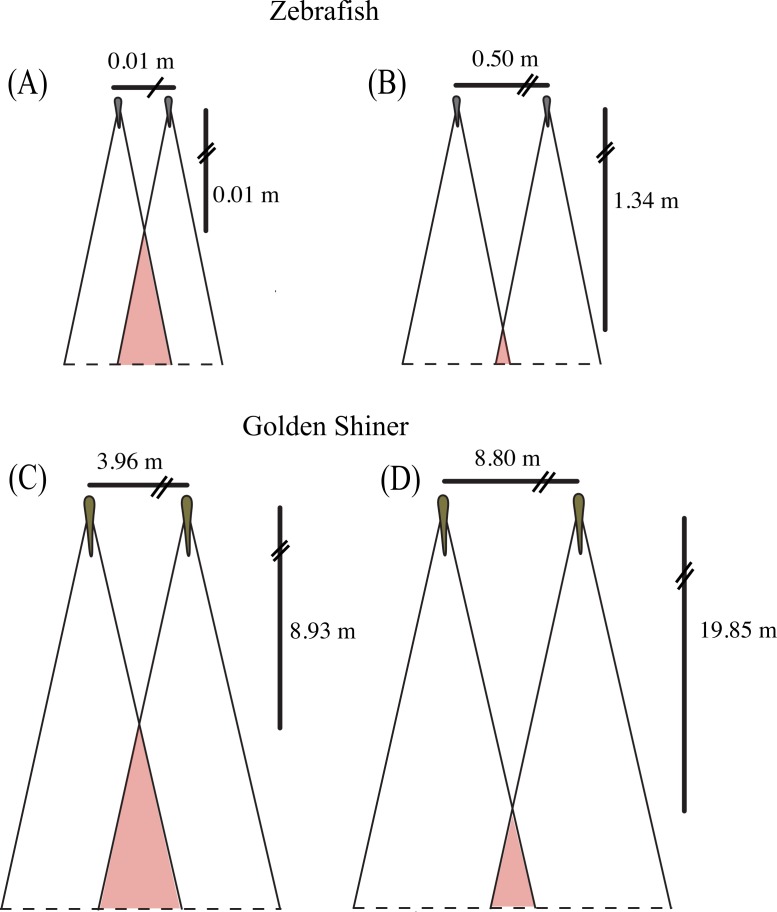
Collective blind area. Schematic representation of the “collective” blind area depicted in red for a hypothetical zebrafish (A, B) and golden shiner school (C, D) viewed from above. Although the width of the blind area does not vary within species, we provide the change in distance to the “collective” blind area using the minimum (A, C) and maximum (B, D) limits their assumed school widths (illustrated for simplicity with two fish) using the equation adopted from Rountree & Sedberry (2009).

Both species have a wide range of visual coverage limited by a posterior blind area, which may make them vulnerable to predator attacks. However, individuals may be able to increase their “collective” visual coverage by associating in groups where the vigilance can be shared among conspecifics (Rountree & Sedberry, 2009). In other words, an individual’s blind area may be compensated for by the visual coverage of its surrounding neighbors, which have the capacity to provide cues to the individual about potential dangers. However, this degree of visual compensation would vary depending on the spatial arrangement of the group members. For example, in a school, where all of the members are facing the same direction, the individual blind areas are spatially located behind the school (Fig. 4). Alternatively, in shoals, group members are not necessarily facing the same direction; therefore, the individual blind areas may project to different regions around the group. In some instances, a “collective” blind area (e.g., red area in Fig. 4) is generated in which all of the individual blind areas overlap, and represents a blind zone that is shared between all of members of the group.

The size and location of the “collective” blind area could have implications for the group’s ability to detect approaching predators (Rountree & Sedberry, 2009). As individuals space themselves out, the distance between the center of the school and the “collective” blind area increases, providing individuals with more time to detect and respond to potential predators approaching from behind (Fig. 4), assuming equal predator attack speeds. Alternatively, as individuals become closer in the group, the distance to the “collective” blind area decreases, reducing the amount of time to respond to a predator attacking from behind (Fig. 4).

Using the blind areas of both cyprinid species reported in this study, the average neighbor separation (Burgess & Shaw, 1981; Miller & Gerlai, 2012) and group sizes observed in the wild (Werner, Hall & Werner, 1978; Pritchard et al., 2001), we calculated the horizontal distance from the center of the school to the “collective” blind area following the equation presented in Rountree & Sedberry (2009). In addition, we used these calculated distance ranges to estimate the time that both species would have available to react to a predator originating from the starting point of the “collective” blind zone (Fig. 4). These estimates assume all individuals are the same size, are oriented in the same direction, and maintain the same nearest neighbor distance.

In the wild, zebrafish are known to form shoals consisting of 2–10 individuals (Pritchard et al., 2001) with nearest neighbor distances ranging from 0.5 to 5.5 cm (Miller & Gerlai, 2012). Based upon these behaviorally measured nearest neighbor distances, we would expect the distance to the “collective” blind area to range from 0.01 to 1.34 m behind the school (Figs. 4A and 4B). Golden shiners have been observed in schools up to 45 individuals with nearest neighbor distances ranging from 9 to 20 cm. Considering this neighbor separation distance, the distance to the “collective” blind area would range from 8.93 to 19.85 m behind the center of a golden shiner school (Figs. 4C and 4D). Considering these relationships, individuals may tradeoff having long interaction distances to increase predator detection with short interaction distances, to allow for the improved resolution of conspecific cues. In fact studies, on zebrafish seem to support this idea as, groups often decrease their neighbor separation immediately following predator exposure (Miller & Gerlai, 2007).

The freshwater garfish (*Xenentodon cancila*) is one of the most prevalent predators of zebrafish (Spence et al., 2008) and are known to rapidly pursue prey (Hossain et al., 2013). We calculated that the zebrafish could potentially increase the time available to react by 13,300% by widening the size of the school from of 0.01 m to its maximum 0.50 m by increasing in neighbor distance from 0.5 to 5.5 cm (0 to 1 body length), which is the range assumed by species in the wild and is also within the range to perceive conspecific cues (Miller & Gerlai, 2012). Largemouth bass (*Micropterus salmoides*) are common predators of golden shiners (Reid, Fox & Whillans, 1999) and by increasing the school width from 3.96 m to 8.80 m with an increase in neighbor distance from 9 to 20 cm (1 to 3 body lengths), golden shiners could potentially increase their reaction time to this predator by 122%. Similarly the neighbor distances used in this calculation represents a range that would allow individuals to perceive conspicuous markings and has also been behaviorally measured in golden shiners (Burgess & Shaw, 1981). In active pursuit predators, like the billfish, which target large schools of prey, increasing the visual range behind the school may improve the survival of the individual group members, as it may allow members to detect rapid changes in the predator’s movements (Hobson, 1979; Pitcher & Parrish, 1993). However, the predator detection benefits of increasing neighbor distance should be outweighed by the costs of reducing the benefits of dilution effects.

**Figure 5:**
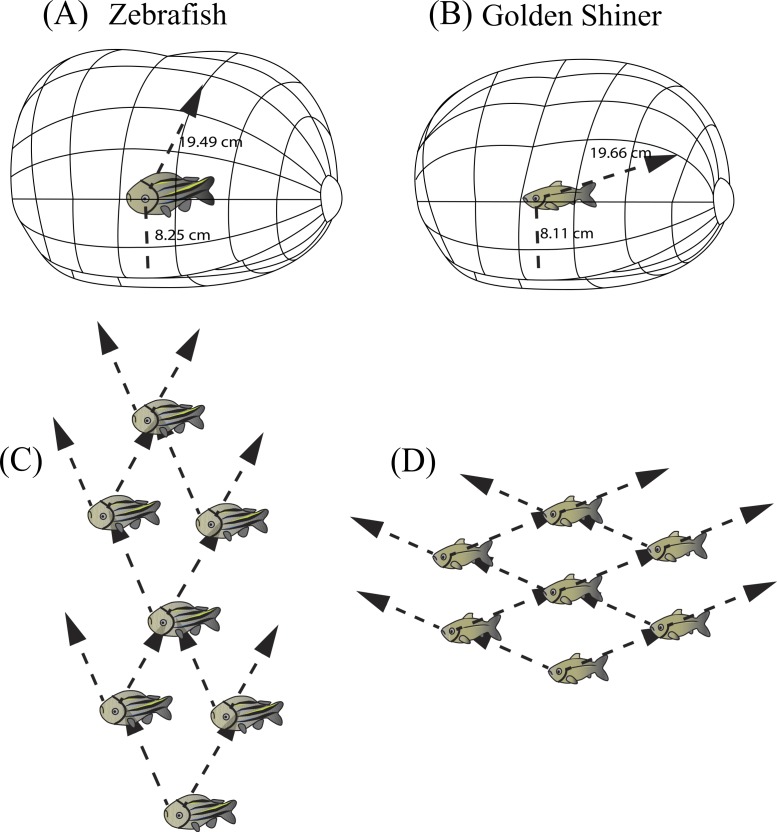
Resolvable distance range and three dimensional school structure. Three-dimensional schematic of the resolvable distance range for the (A) zebrafish and (B) golden shiner based on the resolution of the conspecific conspicuous markings (i.e., zebrafish striping pattern and golden shiner scale width respectively). The arrow represents the projection of the center of acute vision into the visual field, figure adopted from Tyrrell & Fernández-Juricic (2015). Additionally, we used the projection of the centers of acute vision in the (C) zebrafish and (D) golden shiner to generate their hypothetical school shapes, with individuals assuming positions where conspecifics may be resolved with the highest resolution.

In both species, the centers of acute vision are directed towards the front-dorsal region of their visual field (Figs. 2C and 2D). When traveling in a school, zebrafish and golden shiners may prefer positions where they can align this high acuity region with conspecifics to enhance group cohesion. Zebrafish may preferentially respond to neighbors located 63° above the frontal plane of the body and 12° left and right of the sagittal plane of each eye; whereas for golden shiners, they may prefer positions where conspecifics are aligned 32° above the frontal plane of the body and 67° left and right of the sagittal plane for the left and right eye (Fig. 5A and 5B). Actually, there is evidence that information flows unidirectionally through fish groups such that individuals receive information and base their movement decisions from neighbors that are directly in front of them (Herbert-Read et al., 2011). Comparing these estimates with the literature, golden shiners have been shown to respond to changes in the directional movement of neighbors that are ahead of them rather than behind (Katz et al., 2011). Assuming that individuals use the center of acute vision to obtain social cues, and considering the between-species differences in the positioning of the *areae*, we can make predictions about the optimal position of individuals in a group from a visual sensory perspective (Figs. 5C and 5D). Taking into consideration the roughness of the school (surface area/volume ratio), we can predict the zebrafish will have a higher roughness value (i.e., more laterally compressed school; Fig. 5C) than the golden shiner (i.e., more vertically compressed school; Fig. 5D). Variations in roughness have been associated with access to oxygen and predator avoidance (Brierley & Cox, 2010). Unfortunately, there are no estimates of roughness for zebrafish and golden shiners in the literature.

Individuals often maintain a particular distance from group mates (i.e., nearest neighbor distance) (Krause & Ruxton, 2002) by using reference markings on the body (i.e., schooling marks, Bone & Moore, 2008). Based on our estimates of spatial resolving power, we calculated the maximum distance range that both cyprinid species would need to maintain in order to resolve visually these species-specific conspicuous markings, and thus remain attached to the group. In zebrafish, the centers of acute vision are capable of resolving the conspecific striping pattern up to 19.49 cm away using the fronto-dorsal region of their visual field, while the retinal periphery up to 8.25 cm away. Consequently, zebrafish would need to maintain a neighbor distance range of approximately 8–20 cm (∼2–5 body lengths) (Fig. 5A). Golden shiners could resolve the cycloid scales up to 19.66 cm away with their centers of acute vision that project to the fronto-dorsal region of the visual field and up to 8.11 cm with the retinal periphery. Therefore, golden shiners would need to maintain a neighbor separation range of also 8–20 cm (∼1–3 body lengths) (Fig. 5B). However, this distance range assumes ideal, full spectrum light conditions, maximum contrast between the conspecific and the background, and does not account for variations in the optical clarity of the water environment; all these factors could potentially shorten this distance. However, considering that both species tend to inhabit shallow, relatively clear waters (i.e., depths up to 30 cm for the zebrafish and <55 cm for the golden shiner), there is likely to be a low degree of attenuation and consequently, a wide range of available wavelengths to perceive conspecifics. Therefore, the interaction ranges provided by our calculations should not be constrained by the depth of the water environment (Sigler & Sigler, 1987; Krause, Godin & Brown, 1996; Spence et al., 2008). Comparing these estimates with the literature, lab experiments conducted on both species suggest that zebrafish maintain an average nearest neighbor separation of approximately 0.5–5.5 cm (∼1 body length), and golden shiners of 9–20 cm (∼1–3 body lengths) (Burgess & Shaw, 1981; Miller & Gerlai, 2012). Although our predicted distance ranges for the resolution of an entire conspecific are well above the behaviorally determined nearest neighbor distances of both species, our ranges based on the resolution of the conspicuous markings are more consistent with their natural behavior, supporting their role as a potential cue used during collective interactions. However, there are alternative explanations to the observed neighbor distances related to hydrodynamic efficiency (Hemelrijk & Hildenbrandt, 2012) and the use of vibrational cues to sense conspecifics via the lateral line (Partridge & Pitcher, 1980).

### Concluding remarks

In the present study, we developed novel *quantitative* predictions for key parameters (position, spacing, orientation) underlying social interactions of zebrafish and golden shiners based on properties of their visual system. Therefore, an understanding of the species-specific features of the visual system allowed us to predict how individuals would position themselves in groups to optimize the transfer of visual social information. These predictions could be tested empirically in the future.

Most importantly, our findings contradict previous modeling assumptions, which assume that fish have a 60° blind zone and weigh information equally from multiple surrounding neighbors of a fixed distance. We found that the blind areas of both species were less than half the assumed width used in many models simulating fish. We also suggest that the maximum distance at which individuals are able to perceive conspecific cues varies according to different regions of the visual field: 2–5 body lengths in the zebrafish and 1–3 body lengths in the golden shiner, with frontal areas providing the longest distances. Additionally, considering our estimate is in accordance with both species’ behaviorally measured nearest neighbor distances (i.e., ∼1 body length of the zebrafish and 1–3 body lengths for the golden shiner) (Burgess & Shaw, 1981; Miller & Gerlai, 2012), it is possible that these species are using cues about changes in conspecific movement through the resolution of the conspicuous markings. It should also be noted that our distance range estimates for the resolution of another conspecific (i.e., 8–18 body lengths for the zebrafish and 16–39 body lengths for the golden shiner) were much higher than the behaviorally determined interaction range (0.5–2.0 body lengths) typically used in individual-based models to specify when an individual becomes aware of another fish. Thus, making strict sensory assumptions in collective behavior models may misrepresent the basis of local interactions and emerging group structure. The overall implication is that the sensory dimensions of a given species are likely to influence the local interactions driving its collective behavior. We suggest that to better understand the mechanisms driving local interactions in groups, more effort should be invested in assessing how the sensory system may shape the spatial ability to gather information from group mates.

## Supplemental Information

10.7717/peerj.1113/supp-1 Appendix S1 Supplementary Material—Appendix 1 Description of the visual field apparatus and ophthalmoscopic reflex techniqur

10.7717/peerj.1113/supp-2 Figure S1 Visual field apparatus The visual field apparatus designed to measure the retinal visual field in fish. (A) front view and (B) side view of the apparatus with circular dial adjustment in view.

10.7717/peerj.1113/supp-3 Figure S2 Close-up of apparatus with fish model in view For accurate measurement, the fish is placed at the center of the apparatus where the head and body are aligned in the horizontal plane, 90°–270°. The circular rotating metal arm has angular coordinates along the outer perimeter, used for determining the width of the visual field along various elevations around the head.

10.7717/peerj.1113/supp-4 Data S1 Acuity and Eye Measurements Raw Data Files

10.7717/peerj.1113/supp-5 Data S2 Center of Acute Vision Coordinates Raw Data Files

10.7717/peerj.1113/supp-6 Data S3 Visual Field Raw Data Files

10.7717/peerj.1113/supp-7 Data S4 Distance to common blind zone raw data files

## References

[ref-1] Aoki I (1980). An analysis of the schooling behavior of fish: internal organization and process. Bulletin of the Ocean Research Institute, University of Tokyo.

[ref-2] Barbaro A, Einarsson B, Birnir B, Sigurõsson S, Valdimarsson H, Pálsson ÓK, Sveinbjörnsson S, Sigurõsson Þ (2009). Modelling and simulations of the migration of pelagic fish. ICES Journal of Marine Science.

[ref-3] Bianco IH, Kampff AR, Engert F (2011). Prey capture behavior evoked by simple visual stimuli in larval zebrafish. Frontiers in Systems Neuroscience.

[ref-4] Bone Q, Moore R (2008). Sensory systems, and communication. Biology of Fishes.

[ref-5] Brierley AS, Cox MJ (2010). Shapes of krill swarms and fish schools emerge as aggregation members avoid predators and access oxygen. Current Biology.

[ref-6] Burgess JW, Shaw E (1981). Effects of acoustico-lateralis denervation in a facultative schooling fish: a nearest-neighbor matrix analysis. Behavioral and Neural Biology.

[ref-7] Butler SR, Fernández-Juricic E (2014). European starlings recognize the location of robotic conspecific attention. Biology Letters.

[ref-8] Cameron DJ, Rassamdana F, Tam P, Dang K, Yanez C, Ghaemmaghami S, Dehkordi MI (2013). The optokinetic response as a quantitative measure of visual acuity in zebrafish. Journal of Visualized Experiments.

[ref-9] Collin SP, Archer SN, Djamgoz BA, Lowe ER, Partridge JC, Vallerga S (1999). Behavioural ecology and retinal cell topography. Adaptive mechanisms in the ecology of vision.

[ref-10] Collin SP, Northcutt GR (1995). The visual system of the Florida Garfish, *Lepisosteus platyrhincus* (Ginglymodi). Brain, Behavior and Evolution.

[ref-11] Collin SP, Pettigrew JD (1988). Retinal ganglion cell topography in teleosts: a comparison between Nissl-stained material and retrograde labelling from the optic nerve. The Journal of Comparative Neurology.

[ref-12] Collin SP, Pettigrew JD (1989). Quantitative comparison of the limits on visual spatial resolution set by the. ganglion cell layer in twelve species of reef teleosts. Brain, Behavior and Evolution.

[ref-13] Collin SP, Shand J, Collin SP, Marshall JN (2003). Retinal sampling and the visual field in fishes. Sensory processing in aquatic environments.

[ref-14] Couzin ID, Krause J (2003). Self-organization and collective behavior in vertebrates. Advances in the Study of Behavior.

[ref-15] Couzin ID, Krause J, James R, Ruxton GD, Franks NR (2002). Collective memory and spatial sorting in animal groups. Journal of Theoretical Biology.

[ref-16] Dall SRX, Giraldeau L-A, Olsson O, McNamara JM, Stephens DW (2005). Information and its use by animals in evolutionary ecology. Trends in Ecology & Evolution.

[ref-17] Easter SS, Johns PR (1974). Horizontal compensatory eye movements in goldfish (Carassius auratus). Journal of Comparative Physiology.

[ref-18] Ehrlich D (1981). Regional specialization of the chick retina as revealed by the size and density of neurons in the ganglion cell layer. The Journal of Comparative Neurology.

[ref-19] Fernández-Juricic E, Gall MD, Dolan T, Tisdale V, Martin GR (2008). The visual fields of two ground-foraging birds, House Finches and House Sparrows, allow for simultaneous foraging and anti-predator vigilance. Ibis.

[ref-20] Fernández-Juricic E, Kowalski V (2011). Where does a flock end from an information perspective? A comparative experiment with live and robotic birds. Behavioral Ecology.

[ref-21] Garza-Gisholt E, Hemmi JM, Hart NS, Collin SP (2014). A comparison of spatial analysis methods for the construction of topographic maps of retinal cell density. PLoS ONE.

[ref-22] Glaser EM, Wilson PD (1998). The coefficient of error of optical fractionator population size estimates: a computer simulation comparing three estimators. Journal of Microscopy.

[ref-23] Guillemain M, Martin GR, Fritz H (2002). Feeding methods, visual fields and vigilance in dabbling ducks (Anatidae). Functional Ecology.

[ref-24] Guthrie DM, Muntz WRA, Pitcher TJ (1993). Role of vision in fish behaviour. Behaviour of Teleost Fishes.

[ref-25] Hall SJ, Wardle CS, MacLennan DN (1986). Predator evasion in a fish school: test of a model for the fountain effect. Marine Biology.

[ref-26] Harpaz R, Schneidman E (2014). Receptive-field like models accurately predict individual zebrafish behavior in a group. Journal of Molecular Neuroscience.

[ref-27] Hemelrijk CK, Hildenbrandt H (2012). Schools of fish and flocks of birds: their shape and internal structure by self-organization. Interface Focus.

[ref-28] Herbert-Read JE, Perna A, Mann RP, Schaerf TM, Sumpter DJT, Ward a JW (2011). Inferring the rules of interaction of shoaling fish. Proceedings of the National Academy of Sciences of the United States of America.

[ref-29] Heuschele J, Mannerla M, Gienapp P, Candolin U (2009). Environment-dependent use of mate choice cues in sticklebacks. Behavioral Ecology.

[ref-30] Hobson ES (1979). Interactions between piscivorous fishes and their prey. Predator-prey systems in fisheries management.

[ref-31] Hogan KE, Laskowski KL (2013). Indirect information transfer: three-spined sticklebacks use visual alarm cues from frightened conspecifics about an unseen predator. Ethology.

[ref-32] Hossain Y, Jewel AS, Rahman M, Haque ABMM, Elbaghdady HAM, Ohtomi J (2013). Life-history traits of the freshwater garfish *Xenentodon cancila* (Hamilton 1822) (Belonidae) in the Ganges river, Northwestern Bangladesh. Sains Malaysiana.

[ref-33] Hughes A (1975). A quantitative analysis of the cat retinal ganglion cell topography. The Journal of Comparative Neurology.

[ref-34] Hughes A (1977). The topography of vision in mammals of contrasting life style: comparative optics and retinal organization. The visual system in vertebrates.

[ref-35] Kajiura SM (2010). Pupil dilation and visual field in the piked dogfish, *Squalus acanthias*. Environmental Biology of Fishes.

[ref-36] Katz Y, Tunstrøm K, Ioannou CC, Huepe C, Couzin ID (2011). Inferring the structure and dynamics of interactions in schooling fish. Proceedings of the National Academy of Sciences of the United States of America.

[ref-37] Keast A, Fox MG (1992). Space use and feeding patterns of an offshore fish assemblage in a shallow mesotrophic lake. Environmental Biology of Fishes.

[ref-38] Krause J, Godin JJ, Brown D (1996). Phenotypic variability within and between fish shoals. Ecology.

[ref-39] Krause J, Ruxton GD (2002). Living in groups.

[ref-40] Land MF, Nilsson D-E (2012). Animal eyes.

[ref-41] Lemasson BH, Anderson JJ, Goodwin RA (2009). Collective motion in animal groups from a neurobiological perspective: the adaptive benefits of dynamic sensory loads and selective attention. Journal of Theoretical Biology.

[ref-42] Lopez U, Gautrais J, Couzin ID, Theraulaz G (2012). From behavioural analyses to models of collective motion in fish schools. Interface Focus.

[ref-43] Luca RM, Gerlai R (2012). In search of optimal fear inducing stimuli: differential behavioral responses to computer animated images in zebrafish. Behavioural Brain Research.

[ref-44] Lythgoe JN, Partridge JC (1989). Visual pigments and the acquisition of visual information. The Journal of Experimental Biology.

[ref-45] Mangrum WI, Dowling JE, Cohen ED (2002). A morphological classification of ganglion cells in the zebrafish retina. Visual Neuroscience.

[ref-46] Martin GR (1984). The visual fields of the tawny owl, Strix aluco L. Vision Research.

[ref-47] Martin GR (1986). The eye of a passeriform bird, the European starling (*Sturnus vulgaris*): eye movement amplitude, visual fields and schematic optics. Journal of Comparative Physiology A.

[ref-48] Martin GR (2007). Visual fields and their functions in birds. Journal of Ornithology.

[ref-49] Martin GR (2014). The subtlety of simple eyes: the tuning of visual fields to perceptual challenges in birds. Philosophical Transactions of the Royal Society of London. Series B, Biological sciences.

[ref-50] Martin GR, Katzir G (1999). Visual fields in short-toed Eagles, *Circaetus gallicus* (Accipitridae), and the function of binocularity in birds. Brain, Behavior and Evolution.

[ref-51] McComb DM, Kajiura SM (2008). Visual fields of four batoid fishes: a comparative study. The Journal of Experimental Biology.

[ref-52] McComb DM, Tricas TC, Kajiura SM (2009). Enhanced visual fields in hammerhead sharks. The Journal of Experimental Biology.

[ref-53] Miklosi A, Andrew RJ (2006). The Zebrafish as a model for behavioral studies. Zebrafish.

[ref-54] Miller N, Gerlai R (2007). Quantification of shoaling behaviour in zebrafish (*Danio rerio*). Behavioural Brain Research.

[ref-55] Miller N, Gerlai R (2012). From schooling to shoaling: patterns of collective motion in zebrafish (*Danio rerio*). PLoS ONE.

[ref-56] Miyazaki T (2014). Retinal ganglion cell topography in juvenile Pacific bluefin tuna *Thunnus orientalis* (Temminck and Schlegel). Fish Physiology and Biochemistry.

[ref-57] Moore BA, Kamilar JM, Collin SP, Bininda-Emonds ORP, Dominy NJ, Hall MI, Heesy CP, Johnsen S, Lisney TJ, Loew ER, Moritz G, Nava SS, Warrant E, Yopak KE, Fernández-Juricic E (2012). A novel method for comparative analysis of retinal specialization traits from topographic maps. Journal of Vision.

[ref-58] Pankhurst NW (1989). The relationship of ocular morphology to feeding modes and activity periods in shallow marine teleosts from New Zealand. Environmental Biology of Fishes.

[ref-59] Pankhurst PM, Pankhurst NW, Montgomery JC (1993). Comparison of behavioural and morphological measures of visual acuity during ontogeny in a teleost fish, *Forsterygion varium*, tripterygiidae (Forster, 1801). Brain, Behavior and Evolution.

[ref-60] Parrish JK, Viscido S V, Grünbaum D (2002). Self-organized fish schools: an examination of emergent properties. The Biological Bulletin.

[ref-61] Partridge BL (1980). The effect of school size on the structure and dynamics of minnow schools. Animal Behaviour.

[ref-62] Partridge B, Pitcher T (1980). The sensory basis of fish schools: relative roles of lateral line and vision. Journal of Comparative Physiology.

[ref-63] Pettigrew JD, Dreher B, Hopkins CS, McCall MJ, Brown M (1988). Peak density and distribution of ganglion cells in the retinae of microchiropteran bats: implications for visual acuity. Brain, Behavior and Evolution.

[ref-64] Pettigrew JD, Manger PR (2008). Retinal ganglion cell density of the black rhinoceros (Diceros bicornis): calculating visual resolution. Visual Neuroscience.

[ref-65] Pitcher TJ, Parrish JK (1993). Functions of shoaling behaviour in teleosts. Behaviour of Teleost Fishes.

[ref-66] Polverino G, Phamduy P, Porfiri M (2013). Fish and robots swimming together in a water tunnel: robot color and tail-beat frequency influence fish behavior. PLoS ONE.

[ref-67] Pritchard V, Lawrence J, Butlin RK, Krause J (2001). Shoal choice in zebrafish, *Danio rerio*: the influence of shoal size and activity. Animal Behaviour.

[ref-68] Reid SM, Fox MG, Whillans TH (1999). Influence of turbidity on piscivory in largemouth bass (*Micropterus salmoides*). Canadian Journal of Fisheries and Aquatic Sciences.

[ref-69] Romey WWL, Vidal JJM (2013). Sum of heterogeneous blind zones predict movements of simulated groups. Ecological Modelling.

[ref-70] Rosenthal GG, Ryan MJ (2005). Assortative preferences for stripes in danios. Animal Behaviour.

[ref-71] Rountree RA, Sedberry GR (2009). A theoretical model of shoaling behavior based on a consideration of patterns of overlap among the visual fields of individual members. Acta Ethologica.

[ref-72] Rowe DM, Denton EJ (1997). The physical basis of reflective communication between fish, with special reference to the horse mackerel, *Trachurus trachurus*. Philosophical Transactions of the Royal Society B: Biological Sciences.

[ref-73] Saverino C, Gerlai R (2008). The social zebrafish: behavioral responses to conspecific, heterospecific, and computer animated fish. Behavioural Brain Research.

[ref-74] Schairer JO, Bennett MV (1986). Changes in gain of the vestibulo-ocular reflex induced by sinusoidal visual stimulation in goldfish. Brain Research.

[ref-75] Schultz K (2004). Golden shiner. Ken Schultz’s field guide to freshwater fish.

[ref-76] Sigler WF, Sigler JW (1987). Golden shiner. Fishes of the great basin: a natural history.

[ref-77] Spence R, Gerlach G, Lawrence C, Smith C (2008). The behaviour and ecology of the zebrafish, *Danio rerio*. Biological Reviews of the Cambridge Philosophical Society.

[ref-78] Sterratt DC, Lyngholm D, Willshaw DJ, Thompson ID (2013). Standard anatomical and visual space for the mouse retina: computational reconstruction and transformation of flattened retinae with the retistruct package. PLoS Computational Biology.

[ref-79] Sumpter D (2010). Collective animal behavior.

[ref-80] Tamura T, Wisby W (1963). The visual sense of pelagic fishes especially the visual axis and accomodation. Bulletin of Marine Science.

[ref-81] Tappeiner C, Gerber S, Enzmann V, Balmer J, Jazwinska A, Tschopp M (2012). Visual acuity and contrast sensitivity of adult zebrafish. Frontiers in Zoology.

[ref-82] Temple SE, Manietta D, Collin SP (2013). A comparison of behavioural (Landolt C) and anatomical estimates of visual acuity in archerfish (*Toxotes chatareus*). Vision Research.

[ref-83] Tyrrell LP, Fernández-Juricic E, Cooper WE, Blumstein DT (2015). Sensory systems and escape behavior. Escaping from predators: an integrative view of escape decisions.

[ref-84] Tyrrell LP, Moore BA, Loftis C, Fernández-Juricic E (2013). Looking above the prairie: localized and upward acute vision in a native grassland bird. Scientific Reports.

[ref-85] Ullmann JFP, Moore BA, Temple SE, Fernández-Juricic E, Collin SP (2012). The retinal wholemount technique: a window to understanding the brain and behaviour. Brain, Behavior and Evolution.

[ref-86] Vicsek T, Czirók A, Ben-Jacob E, Cohen I, Shochet O (1995). Novel type of phase transition in a system of self-driven particles. Physical Review Letters.

[ref-87] Walls GL (1942). The vertebrate eye and its adaptive radiation.

[ref-88] Watanuki N, Kawamura G, Kaneuchi S, Iwashita T (2000). Role of vision in behavior, visual field, and visual acuity of cuttlefish *Sepia esculenta*. Fisheries Science.

[ref-89] Werner EE, Hall DJ, Werner MD (1978). Littoral zone fish communities of two Florida lakes and a comparison with Michigan lakes. Environmental Biology of Fishes.

[ref-90] Williams DR, Coletta NJ (1987). Cone spacing and the visual resolution limit. Journal of the Optical Society of America A.

[ref-91] Wilson JM, Bunte RM, Carty AJ (2009). Evaluation of rapid cooling and tricaine methanesulfonate (MS222) as methods of euthanasia in zebrafish (*Danio rerio*). Journal of the American Association for Laboratory Animal Science.

[ref-92] Wood AJ, Ackland GJ (2007). Evolving the selfish herd: emergence of distinct aggregating strategies in an individual-based model. Proceedings. Biological Sciences/The Royal Society.

