# Supplementary material for: Vision in two cyprinid fish: implications for collective behavior
Source: PeerJ. 2015 Aug 4;3:e1113. doi: 10.7717/peerj.1113 (PMC4540049; doi:10.7717/peerj.1113)
Supplement: Appendix S1 — Description of the visual field apparatus and ophthalmoscopic reflex techniqur [file peerj-03-1113-s001.docx]

**Appendix 1: Visual field configuration and apparatus design**

We scaled down the visual field apparatus design of Martin (1984) to allow for the observation of the retinal reflex with an ophthalmoscope. This was accomplished by reducing the diameter of circular rotating arm to 24 cm. The apparatus was configured such that 90° and 270° were directly in front and behind of the animal, respectively, along the horizontal plane, while 0° and 180° represented the vertical plane above and below the animal, respectively (Figs. 2 c, d).

Fish were measured immediately after euthanasia and were fixed on top of two metal prongs located at the center of the coordinate system of the apparatus (Fig. 2). We maintained the position of the animal such that the body was parallel to the ground (i.e. 90° horizontal plane). The adjustment dial on the side of the apparatus allowed the observer to measure the visual field across various elevations about the animal (Fig. 2). We recorded the visual field configuration across all angles around the head along 10° increments in elevation. The elevations 160°-270° were obtained such that the dorsal side of the fish was 0° and the ventral side was 180° (Fig. 2). Complete rotation of the circular arm was obstructed along the elevations of 170°-260°; in order to record the visual field along this region, we rotated the body of the animal along the x-axis, 180° such that the dorsal side of the fish faced 180° and the ventral side faced 0°.
