# Supplementary figures and images for: Vision in two cyprinid fish: implications for collective behavior
Source: PeerJ. 2015 Aug 4;3:e1113. doi: 10.7717/peerj.1113 (PMC4540049; doi:10.7717/peerj.1113)

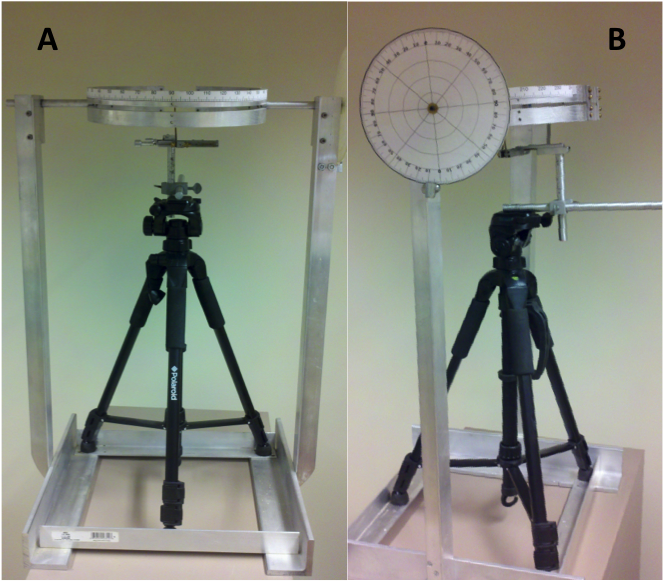

Supplement: Figure S1 — The visual field apparatus designed to measure the retinal visual field in fish. (A) front view and (B) side view of the apparatus with circular dial adjustment in view. [file peerj-03-1113-s002.png]

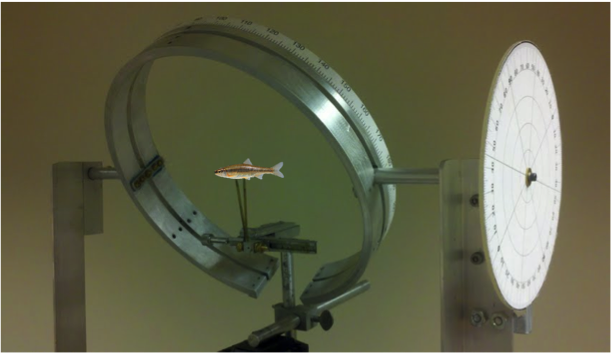

Supplement: Figure S2 — For accurate measurement, the fish is placed at the center of the apparatus where the head and body are aligned in the horizontal plane, 90°–270°. The circular rotating metal arm has angular coordinates along the outer perimeter, used for determining the width of the visual field along various elevations around the head. [file peerj-03-1113-s003.png]
